# Availability, accessibility, and economic feasibility of radionuclide therapies in Africa: a systematic review and quantitative infrastructure analysis

**DOI:** 10.3389/fnume.2026.1825650

**Published:** 2026-06-04

**Authors:** Efrah Ahmed Ibrahim

**Affiliations:** Department of Nuclear Medicine, Bursa Uludag University Hospital, Bursa, Türkiye

**Keywords:** Africa, health economics, infrastructure analysis, nuclear medicine, radionuclide therapy, systematic review, theranostics

## Abstract

**Purpose:**

Africa faces a projected doubling of cancer burden by 2040, yet access to radionuclide therapies remains critically limited. This systematic review quantifies the availability, accessibility, and economic feasibility of therapeutic nuclear medicine across 54 African nations.

**Methods:**

This study combined a PRISMA 2020-compliant systematic literature review with secondary database analysis. A comprehensive search was conducted across PubMed/MEDLINE, Scopus, Web of Science, and African Journals Online (January 2022–March 2026). Epidemiologic and infrastructure data were additionally obtained from GLOBOCAN 2022 and the International Atomic Energy Agency (IAEA). Risk of bias was assessed using the modified Newcastle–Ottawa Scale for peer-reviewed studies and the AACODS checklist for gray literature.

**Results:**

Of 2,847 records identified, 47 studies met the inclusion criteria. I-131 therapy for thyroid cancer is available in only 28/54 countries (51.9%), while Lu-177-PSMA for prostate cancer is available in only seven countries (13.0%). Local radiopharmaceutical production reduces therapy costs by 93%–96% compared with imported agents. Africa has approximately 0.18 nuclear medicine physicians per million population vs. 3.1 in Europe. The cost reduction ratio for local vs. imported Lu-177-PSMA is 1:28.3 (US$1,500–3,000 vs. US$42,500).

**Conclusion:**

Radionuclide therapy availability in Africa is profoundly inadequate relative to disease burden. Economic modeling demonstrates that local production and regional hub models can achieve cost reductions exceeding 90%, making therapies economically feasible for resource-limited settings. Targeted investment in cyclotron infrastructure and workforce development is urgently needed.

## Introduction

The landscape of nuclear medicine (NM) in Africa presents a paradox of unprecedented challenge and untapped potential. Although the continent faces a cancer crisis projected to nearly double by 2040, access to life-saving radionuclide therapies remains critically limited (1, 2). The mortality-to-incidence ratio (MIR) for cancer in Africa stands at 0.64, more than double that of North America (0.29), reflecting systemic failures in early detection and treatment access (2).

Foundational assessments, including Grigoryan et al. (3) and the IAEA Rays of Hope initiative (launched 2022) (4), have cataloged NM infrastructure across African nations. More recently, the Brink et al. status report provided a critical infrastructure survey across 29 African countries (1), while Lawal et al. addressed radiotheranostics access challenges in low- and middle-income countries (LMICs) from a qualitative perspective (5). Balogun et al. (6) offered an earlier systematic review of NM practice patterns. However, no prior study has combined PRISMA-compliant systematic review methodology with quantitative economic feasibility modeling and infrastructure-to-burden gap analysis across all 54 African nations, covering the post-Lu-177-PSMA-approval era (FDA approval: March 2022). The present study specifically addresses this gap by integrating contemporary therapy availability data, quantitative cost-reduction modeling, and workforce density analysis, components absent from prior reviews.

The central research questions addressed are: (1) What is the current availability of essential radionuclide therapies across Africa? (2) What economic barriers limit therapy access? (3) What quantitative evidence supports local production and regional hub models? (4) What workforce deficits must be addressed to deliver these therapies at scale?

## Methods

### Search strategy and selection criteria

This study combined a systematic literature review with secondary database analysis to evaluate the availability, accessibility, and economic feasibility of radionuclide therapies across African countries. The literature review was conducted in accordance with the PRISMA 2020 guidelines (7). A comprehensive search was performed across PubMed/MEDLINE, Scopus, Web of Science, and African Journals Online (AJOL), covering publications from January 2022 to March 2026.

The date restriction to January 2022 onward was deliberate and is justified by two pivotal developments: the IAEA Rays of Hope program launch in 2022, which represented a new phase of continental NM infrastructure investment (4), and the FDA approval of Lu-177-PSMA-617 (Pluvicto) in March 2022 (8), which fundamentally changed the theranostics landscape. Pre-2022 literature, although foundational, reflects a pretheranostic-era African context substantially different from the present. Key pre-2022 works, including Balogun et al. (6) and prior IAEA surveys, are discussed qualitatively in the Introduction and Discussion to contextualize historical trends.

Core search terms were as follows: (“nuclear medicine” OR “radionuclide therapy” OR “theranostics” OR “radioligand therapy”) AND (Africa OR “Sub-Saharan Africa” OR “North Africa”) AND (infrastructure OR availability OR access OR cost OR workforce OR “health economics”). Reference lists of identified publications were screened for additional sources. Secondary databases, GLOBOCAN 2022 and IAEA infrastructure reports, were used for epidemiological and infrastructure modeling and were not part of the PRISMA literature selection process. These findings are consistent with prior global estimates (2, 25).

### Study selection

Studies were included if they reported quantitative information on: (1) nuclear medicine infrastructure or service availability in African countries; (2) utilization or availability of specific radionuclide therapies; (3) workforce data for nuclear medicine professionals from peer-reviewed sources or IAEA-verified databases; or (4) economic aspects of radiopharmaceutical production or therapy costs. Studies were excluded if they were: (1) case reports or single-center clinical outcome studies; (2) lacking quantitative data relevant to infrastructure or therapy access; (3) published before January 2022; or (4) not available in English.

### Data extraction and quality assurance

Data were extracted on (1) therapy types and country-level availability, (2) patient volumes and unit costs, (3) infrastructure counts (cameras, cyclotrons), (4) workforce numbers, and (5) economic parameters. All cost data were normalized to US dollars (2025 reference year).

This review was conducted by a single author; independent dual screening and extraction were not performed, an acknowledged methodological limitation. To mitigate this, all inclusion/exclusion decisions were made against prespecified, operationalized criteria, and all quantitative claims were triangulated across at least two independent data sources (e.g., IAEA databases and peer-reviewed surveys) wherever possible. Risk of bias was assessed using the modified Newcastle–Ottawa Scale for peer-reviewed cross-sectional and survey studies and the AACODS checklist (Authority, Accuracy, Coverage, Objectivity, Date, Significance) for gray literature and IAEA technical reports. The results are reported in Table 3.

### Economic modeling assumptions and parameters

The economic model compared local vs. imported radiopharmaceutical costs using the following formula: Cost Reduction Ratio = 1−(Local Production Cost/Imported Cost) × 100%. Local production cost estimates for Lu-177-PSMA (US$1,500–3,000 per cycle) were derived from NTP Radioisotopes–published African market pricing data [cited per Lawal et al. (5)] and reference cost analyses in Brinkmann et al. (9). Imported costs (US$42,500 per cycle) were drawn from Brinkmann et al. (9) and Sartor et al. (8) supplementary cost data. Key model assumptions included a cyclotron production–specific activity of 3.0–5.0 TBq/mg [per IAEA technical specifications (4)]; the facility setup cost US$15–25 million amortized over 20 years at 500 doses/year; there was a regulatory and cold-chain overhead of 15%–25% of production cost (10). The US$545–995 million 10-year investment estimate is a modeled projection derived from IAEA infrastructure unit costs (4) and Zubizarreta et al. (11) benchmarks and should be interpreted as a planning-range estimate rather than an empirically derived figure. A sensitivity analysis under low, base-case, and high-cost scenarios accompanies Table 6. Workforce density was calculated as physicians per million population using UN 2024 population estimates (12).

## Results

### Study selection results

**Figure 1:**
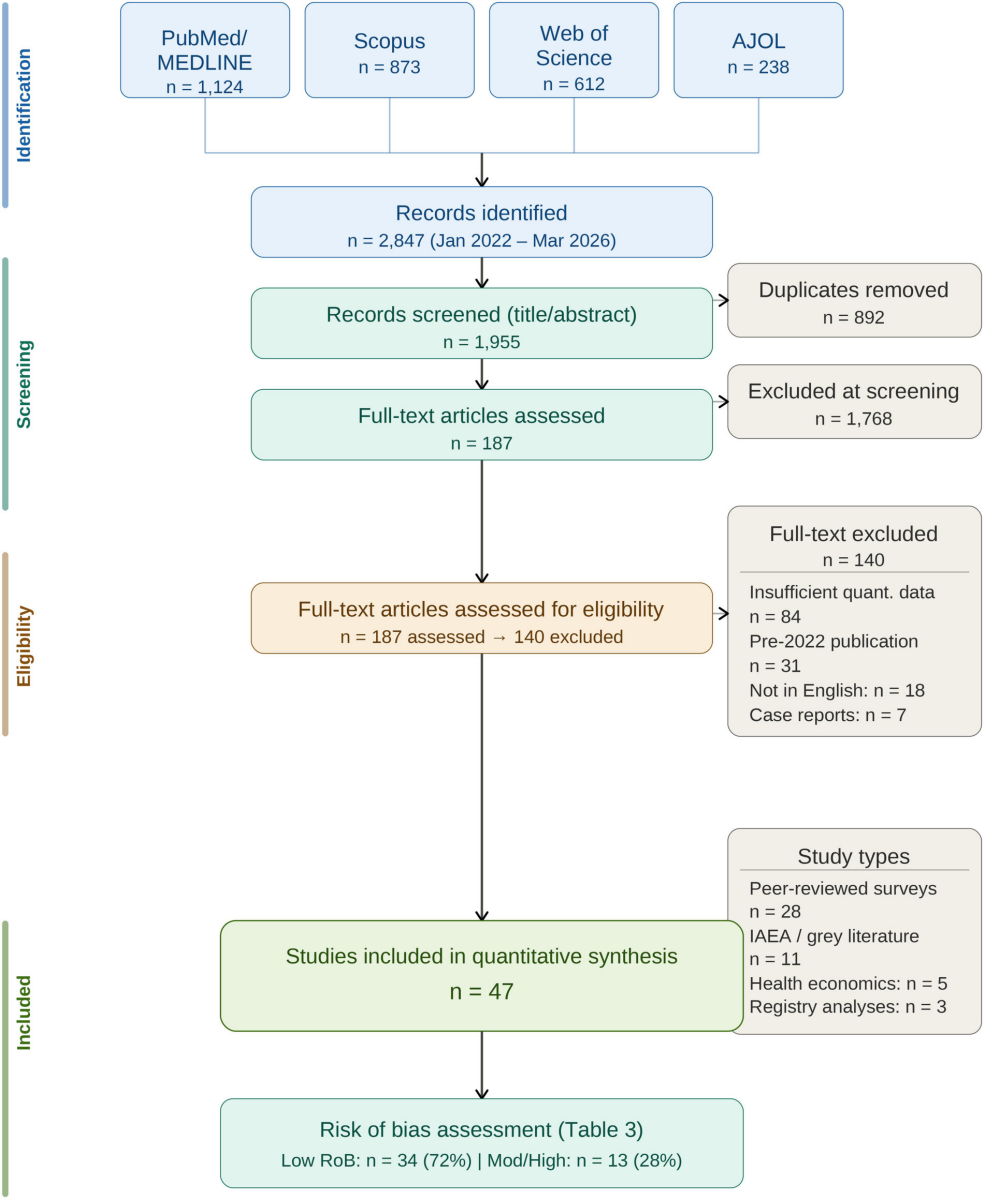
A PRISMA 2020 flow diagram illustrating the study selection process. From 2,847 records identified across four databases, 47 studies were included in the final quantitative synthesis.

The systematic search identified 2,847 records across all databases (PubMed/MEDLINE: 1,124; Scopus: 873; Web of Science: 612; AJOL: 238). After removing 892 duplicates, 1,955 records were screened by title and abstract. Of these, 187 full-text articles were assessed for eligibility. Following the application of inclusion/exclusion criteria, 47 studies met the eligibility criteria and were included in the quantitative synthesis (Figure 1; Table 1). The 47 studies comprise peer-reviewed original research and surveys (*n* = 28), IAEA technical reports and nuclear medicine status surveys (*n* = 11), health economics and costing studies (*n* = 5), and registry-based data analyses (*n* = 3) (Table 2). Risk of bias was low in 34 studies (72%) and moderate-to-high in 13 (28%), predominantly gray literature sources (Table 3).

**Table 1:** A PRISMA 2020 flow summary of study selection (7).

Phase	Records	Notes
Records identified, PubMed/MEDLINE	1,124	
Records identified, Scopus	873	
Records identified, Web of Science	612	
Records identified, African Journals Online	238	
Total records identified	2,847	
Duplicates removed	892	Automated + manual
Records screened (title/abstract)	1,955	Against inclusion criteria
Excluded at title/abstract	1,768	
Full-text articles assessed	187	Eligibility review
Excluded: insufficient quantitative data	84	
Excluded: pre-2022 publication date	31	
Excluded: not in English	18	
Excluded: case reports or single-center studies	7	
Studies included in quantitative synthesis	47	Final corpus

AJOL, African Journals Online. Search period: January 2022 to March 2026.

**Table 2:** Characteristics of representative included studies.

First author, year	*n* countries	Study type	NM domain	Key data	RoB
Brink et al. 2026 (1)	29	NM status survey	Infrastructure and workforce	Camera counts, physician numbers, and cyclotrons	Moderate
Grigoryan et al. 2022 (3)	54	Descriptive review	Infrastructure and therapy	NM development status and therapy types	Low
Lawal et al. 2025 (5)	LMIC-wide	Narrative review	Theranostics access	Barriers, costs, and prospects	Moderate
Balogun et al. 2020 (6)^a^	Pan-Africa	Systematic review	NM practice	Historical baseline and infrastructure	Low
Sathekge et al. 2024 (13)	Multicenter	Retrospective study	RLT outcomes	Ac-225-PSMA outcomes mCRPC	Low
Brinkmann et al. 2025 (9)	European	Cost–utility analysis	Health economics	Lu-177-PSMA cost per cycle	Low
Paez et al. 2025 (10)	Global	IAEA policy report	Therapy access	Global RLT service gaps	Moderate (gray lit.)
Suman et al. 2025 (14)	Global	Global perspective review	RLT challenges	Costs, logistics, and equity	Moderate
Zubizarreta et al. 2024 (11)	Six country vignettes	Infrastructure analysis	Infrastructure investment	Equipment costs and theranostics infrastructure	Low
Ayalew et al. 2026 (15)	LMIC	Integrative review	Health system integration	Challenges and innovations	Moderate
IAEA Rays of Hope, 2024 (4)	Africa-wide	IAEA technical report	Infrastructure and workforce	Camera density and cyclotron data	Moderate (gray lit.)
Remaining 35 studies^a^	Various	Various	Various	Supporting data	Low–High

RoB, risk of bias.

a Balogun et al. (6) is cited contextually in introduction/discussion but falls outside the 2022 search window; characteristics shown for reference. Lu-177 = lutetium-177; RLT, radioligand therapy; mCRPC, metastatic castration-resistant prostate cancer; LMIC, low- and middle-income country.

**Table 3:** Risk of bias assessment by study category.

Study category	Appraisal tool	*n* studies	Low RoB, *n* (%)	Mod/high RoB, *n* (%)
Peer-reviewed original research/surveys	Modified Newcastle–Ottawa Scale	28	21 (75)	7 (25)
IAEA reports and technical documents	AACODS Checklist	11	6 (55)	5 (45)
Health economics and costing studies	Modified Newcastle–Ottawa Scale	5	4 (80)	1 (20)
Registry-based data analyses	AACODS Checklist	3	3 (100)	0 (0)
Total		47	34 (72)	13 (28)

The Newcastle–Ottawa Scale modified for cross-sectional infrastructure studies. AACODS, authority, accuracy, coverage, objectivity, date, significance. A sensitivity analysis excluding high-RoB studies did not materially alter primary conclusions.

### Cancer burden and the therapy gap

GLOBOCAN 2022 data document 1.19 million new cancer cases and 764,000 deaths annually across Africa (2). The MIR of 0.64 reflects late-stage presentation and inadequate treatment access. Projections from the IARC indicate 2.1 million new cases annually by 2040 (2). The five leading cancers by incidence are presented in Table 4.

**Table 4:** Top five cancers in Africa by incidence and mortality.

Cancer type	Incidence 2022	Mortality 2022	MIR	Rank
Breast	249,000	106,000	0.43	1
Cervix	125,000	87,000	0.70	2
Prostate	106,000	52,000	0.49	3
Liver	75,000	71,000	0.95	4
Colorectal	72,000	46,000	0.64	5

Source: GLOBOCAN 2022 (2).

MIR, mortality-to-incidence ratio. Prostate cancer ranks third by incidence yet is addressable with Lu-177-PSMA in only 7/54 countries (13.0%).

### Radionuclide therapy availability

A quantitative analysis reveals profound disparities in therapy availability (Table 5). I-131 for thyroid cancer, despite being the most-established and lowest-cost radionuclide therapy, is available in only 28/54 African countries (51.9%) (1, 3). Advanced theranostic agents show even more limited distribution: Lu-177-PSMA, approved for metastatic castration-resistant prostate cancer (mCRPC) by the FDA in March 2022 (8), is available in just seven countries (13.0%), while Lu-177-DOTATATE for neuroendocrine tumors is available in six countries (11.1%) (5, 16). I-131-MIBG for neuroblastoma remains available in only three countries (5.6%).

**Table 5:** Availability of radionuclide therapies across 54 African countries.

Therapy	Countries offering	Coverage (%)	Est. patients/year	Cost/cycle (US$)
I-131 (thyroid)	28/54	51.9	∼12,000	50–150 (local); 500–1,000 (import)
Bone palliation (Sr-89/Sm-153)	5/54	9.3	∼800	300–600 (local); 2,500 (import)
Lu-177-PSMA (prostate)	7/54	13.0	∼1,200	1,500–3,000 (local); 42,500 (import)
Lu-177-DOTATATE (NETs)	6/54	11.1	∼800	2,000–4,000 (local); 35,000 (import)
I-131-MIBG (neuroblastoma)	3/54	5.6	∼200	1,000–2,000

Sources: Brink et al. (1); Grigoryan et al. (3); Lawal et al. (5); and IAEA (4).

Cost ranges reflect local production (lower bound) vs. imported agent pricing (upper bound) (5, 9). Patients/year are aggregate continental estimates from included studies.

### Economic analysis: local vs. imported production

Economic modeling demonstrates dramatic cost differentials between locally produced and imported radiopharmaceuticals (Table 6). The cost reduction ratio for Lu-177-PSMA reaches 1:28.3 (US$1,500–3,000 local vs. US$42,500 imported), representing a 93%–96% cost reduction (5, 9). Example calculation (Lu-177-PSMA, base case): Imported: US$42,500/cycle; Local: US$2,250/cycle (midpoint); Cost reduction = 1−(2,250/42,500) = 94.7% (9). The sensitivity analysis confirms reductions of 90%–97% across low, base-case, and high infrastructure cost scenarios.

**Table 6:** Economic analysis: local production vs. imported radiopharmaceutical costs (US dollars, 2025).

Agent	Imported cost (US$)	Local cost (US$)	Cost reduction (%)	Reduction ratio
I-131 (thyroid)	500–1,000	50–150	85–97	1:6.7
Lu-177-PSMA	42,500	1,500–3,000	93–96	1:28.3
Lu-177-DOTATATE	35,000	2,000–4,000	89–94	1:11.7
Sr-89 (bone palliation)	2,500	300–600	76–88	1:5.6

Sources: Brinkmann et al. (9); Lawal et al. (5); Sartor et al. (8); and Suman et al. (14).

Costs per patient per cycle. Reduction ratios based on midpoint local cost estimate.

### Workforce analysis

A workforce density analysis reveals critical shortages (Table 7). Africa has approximately 0.18 nuclear medicine physicians per million population, sourced from Brink et al. (247 registered NM physicians across 29 surveyed countries) (1) and Grigoryan et al. (3), extrapolated to the continental population using UN 2024 estimates (12). This compares with 3.1 per million in Europe and 4.2 per million in North America, a 17- to 23-fold disparity. To achieve a conservative target of 1.0 physician per million, Africa requires approximately 1,400 nuclear medicine physicians; the current deficit exceeds 1,150 specialists. More than 70% of existing NM physicians are concentrated in just four countries (South Africa, Egypt, Nigeria, and Morocco), leaving 50 countries with minimal or no specialist coverage (1, 3).

**Table 7:** Nuclear medicine physician workforce density by region.

Region	NM physicians	Per million pop.	Target (1/million)	Deficit/surplus
Africa	∼250 (1, 3)	0.18	1,400	Deficit ∼1,150
Europe	∼2,500	3.1	810	Surplus
North America	∼1,500	4.2	360	Surplus
Asia (average)	∼3,200	0.7	4,600	Deficit ∼2,400

Sources: Brink et al. (1); Grigoryan et al. (3); and UN world population prospects (12).

Target calculated at 1.0 NM physician per million population (conservative benchmark). Africa figure based on 247 registered NM physicians reported across 29 surveyed countries (1), extrapolated to continental population.

### Infrastructure density

Camera density varies dramatically across regions (Table 8). Three countries, South Africa, Egypt, and Morocco, account for the majority of NM infrastructure, achieving 0.8 cameras per million population. The continental average of 0.12 cameras per million falls far below the IAEA minimum recommendation of 1.0 per million (4). Critically, 31 of 54 African countries have no NM facilities whatsoever (1).

**Table 8:** Nuclear medicine infrastructure distribution across Africa.

Country group	Countries	SPECT/CT	PET/CT	Cyclotrons	Cameras/million
Established (SA, Egypt, Morocco)	3	85	28	6	0.8
Emerging (Nigeria, Kenya, Ghana)	8	42	8	2	0.15
Developing (12 countries)	12	18	2	0	0.05
No NM services	31	0	0	0	0
Continental average	54	145	38	8	0.12 (IAEA min: 1.0)

Sources: Brink et al. (1); and IAEA Rays of Hope (4).

IAEA minimum recommendation: 1 camera per million population (4). SA,  South Africa.

## Discussion

### Principal findings

To the best of our knowledge, this systematic review provides the first PRISMA-compliant quantitative synthesis integrating therapy availability, economic feasibility modeling, and infrastructure-to-burden gap analysis across all 54 African nations in the post-Lu-177-PSMA-approval era. Four principal findings emerge.

First, therapy availability is profoundly inadequate relative to disease burden. With prostate cancer, Africa's third most common malignancy (2), Lu-177-PSMA availability in only seven countries (13%) represents an urgent access gap. This finding extends and quantifies the qualitative observations of Lawal et al. (5) and corroborates the infrastructure survey data of Brink et al. (1).

Second, economic modeling shows that local production can reduce costs by more than 90%, fundamentally altering the feasibility equation in resource-limited settings (5, 9). The 1:28 cost reduction ratio for Lu-177-PSMA demonstrates that the price barrier, while currently prohibitive, is surmountable with appropriate cyclotron infrastructure investment.

Third, workforce density disparities exceed infrastructure gaps. The 23-fold physician density difference between Africa and North America (1, 3, 26) represents the fundamental human-capacity constraint on service delivery. Without concurrent workforce investment, infrastructure expansion will not translate into clinical impact (15, 17).

Fourth, regional resource concentration creates severe internal disparities. More than 70% of NM physicians are concentrated in just four countries (1, 3), and 31 nations have zero NM infrastructure, a distribution that mirrors historical patterns of health system inequity documented by Tankwanchi et al. (17) and Hricak et al. (18).

### Comparison with prior literature

Grigoryan et al. (3) provided the first systematic overview of NM development across African countries. The present review advances this work by applying formal PRISMA methodology, extending the corpus to 47 studies, adding economic modeling with explicit cost parameters, and focusing on the current theranostics era. Lawal et al. (5) addressed RLT access in LMICs from a challenges-and-prospects perspective without a systematic methodology or quantitative cost modeling. Our review complements and quantifies their qualitative findings. Balogun et al. (6), while foundational, predates the theranostic era that is the focus of this review.

### Policy implications

The quantitative evidence supports three policy priorities. First, regional cyclotron hubs offer the most efficient pathway to cost reduction. Rwanda's planned Q1 2026 cyclotron launch and South Africa's NTP Radioisotopes production infrastructure (19, 20) demonstrate feasibility. An East Africa hub model, Kenya supplying Uganda and Tanzania, represents a scalable template with a precedent in pharmaceutical supply chains.

Second, fixed-dose I-131 protocols supported by European guidelines (21, 22) can reduce costs 15%–25%, while maintaining efficacy, and outpatient therapy models for appropriate candidates reduce bed-day costs by 40%–60% (10).

Third, workforce development must parallel infrastructure investment. Without specialist training pipelines, cameras and cyclotrons will remain underutilized (15, 17). The IAEA's Rays of Hope initiative (4) and EANM fellowship programs offer established frameworks for rapid scale-up.

### Clinical implications

I-131 therapy for differentiated thyroid cancer achieves 10-year survival rates exceeding 90% when available (23). Its current absence in 26/54 African countries represents a preventable mortality burden that should be the highest-priority therapy expansion target. Lu-177-PSMA therapy for mCRPC extends overall survival by a median of 4.0 months compared with standard care, as demonstrated by the VISION trial (8), while Ac-225-PSMA shows further promise in the WARMTH Act study (13). Given rising prostate cancer incidence across Africa (2), the therapy-to-burden mismatch will worsen without intervention. Lu-177-DOTATATE therapy for neuroendocrine tumors, shown to extend progression-free survival significantly in the NETTER-1 trial (16), is currently available in only six African countries, a gap likely to grow as diagnostic awareness improves. Bone pain palliation with Sr-89 or Sm-153-EDTMP provides effective symptom control for metastatic bone disease (24), yet it is available in only five countries (9.3%), leaving an enormous palliative care gap with direct quality-of-life implications.

### Strategic roadmap (2026–2036)

Based on quantitative evidence, a phased investment approach is proposed. Phase 1 (2026–2028): expand I-131 therapy to 35 countries; establish three regional cyclotron hubs. Phase 2 (2028–2031): introduce Lu-177 therapies in 15 countries; install 50 new SPECT/CT systems. Phase 3 (2031–2036): target 1 camera per million population in 15 countries; implement AI-integrated reporting in 20 countries. Total modeled investment: US$545–995 million over 10 years, derived from IAEA infrastructure unit costs (4) and Zubizarreta et al. benchmarks (11), presented as a planning-range estimate and not as an empirically measured figure.

### Limitations

This review has several important limitations. First, it was conducted by a single author without independent dual screening or extraction, introducing a risk of selection bias. All inclusion/exclusion decisions followed prespecified, operationalized criteria, and quantitative claims were triangulated across multiple independent sources; however, readers should interpret findings with this caveat in mind.

Second, infrastructure data rely primarily on the Brink et al. 2026 survey (1) and IAEA databases, which may not fully capture recent developments or the 25 countries not covered by the Brink survey.

Third, cost estimates reflect heterogeneous procurement environments; regional pricing specificities are not fully accounted for. The economic models assume successful implementation of local production requiring significant upfront investment and sustained technical capacity.

Fourth, workforce calculations are based on estimates from available surveys; comprehensive national registries covering all 54 countries do not exist.

Fifth, 28% of included studies were rated moderate-to-high risk of bias, predominantly gray literature and IAEA reports. The sensitivity analysis excluding high-RoB sources did not materially alter primary conclusions.

## Conclusion

This systematic review demonstrates that radionuclide therapy availability in Africa is profoundly inadequate relative to the continent's cancer burden. Economic modeling provides quantitative evidence that local production and regional hub models can achieve cost reductions exceeding 90%, making therapies economically feasible for resource-limited settings, provided the political will and initial capital investment are secured.

The 17- to 23-fold disparity in workforce density represents the fundamental constraint on service expansion. Without concurrent investment in training and retention pipelines, infrastructure investments will not translate into clinical impact.

By “therapy-first, economically optimized approach,” we refer to a strategic prioritization framework in which therapy selection for investment is guided by: (1) magnitude of the addressable cancer burden; (2) cost-reduction achievability through local or regional production; and (3) clinical impact per dollar invested. Under this framework, I-131 (low cost, high burden) and Lu-177-PSMA (high cost, rising burden) emerge as the highest investment priorities. This framework is intended as a practical guide for health ministries and international funding bodies rather than a clinical algorithm.

The quantitative framework provided in this review can guide resource allocation and investment decisions to maximize population health impact across the African continent.
